# Biological Synthesis of Silver Nanoparticles and Prospects in Plant Disease Management

**DOI:** 10.3390/molecules27154754

**Published:** 2022-07-25

**Authors:** Moh Tariq, Khan Nazima Mohammad, Bilal Ahmed, Mansoor A. Siddiqui, Jintae Lee

**Affiliations:** 1 Department of Botany, Lords University, Alwar 301028, India; 2 Section of Plant Pathology and Nematology, Aligarh Muslim University, Aligarh 202002, India; khannazima160@gmail.com (K.N.M.); mansoor_bot@yahoo.co.in (M.A.S.); 3 School of Chemical Engineering, Yeungnam University, Gyeongsan 38541, Korea; jtlee@ynu.ac.kr

**Keywords:** silver nanoparticles, sustainable agriculture, plant disease, pathogen, green synthesis

## Abstract

Exploration of nanoparticles (NPs) for various biological and environmental applications has become one of the most important attributes of nanotechnology. Due to remarkable physicochemical properties, silver nanoparticles (AgNPs) are the most explored and used NPs in wide-ranging applications. Also, they have proven to be of high commercial use since they possess great chemical stability, conductivity, catalytic activity, and antimicrobial potential. Though several methods including chemical and physical methods have been devised, biological approaches using organisms such as bacteria, fungi, and plants have emerged as economical, safe, and effective alternatives for the biosynthesis of AgNPs. Recent studies highlight the potential of AgNPs in modern agricultural practices to control the growth and spread of infectious pathogenic microorganisms since the introduction of AgNPs effectively reduces plant diseases caused by a spectrum of bacteria and fungi. In this review, we highlight the biosynthesis of AgNPs and discuss their applications in plant disease management with recent examples. It is proposed that AgNPs are prospective NPs for the successful inhibition of pathogen growth and plant disease management. This review gives a better understanding of new biological approaches for AgNP synthesis and modes of their optimized applications that could contribute to sustainable agriculture.

## 1. Introduction

Phytopathogenic microbes and plant pests are natural habitants of the plant’s surrounding environment and cause huge crop losses with a worldwide estimated reduction in crop production of 20–40% on an annual basis [1]. Applications of pesticides including fungicides and insecticides are currently being used [2] on a very large scale and are considered a key to pest management. The use of chemical pesticides is a rapid killing strategy against pathogenic microorganisms; however, their overuse and long-term persistence in soils unwantedly reduce soil fertility [3] and disrupt the natural micro and macro soil biota. Among them, many plant-beneficial microbes are either killed or their natural ecology is compromised. This necessitates the development of alternative strategies to control plant diseases. In this regard, nanotechnology seems to be an alternative; it has also progressed significantly in the field of medicine, e.g., as alternative antimicrobials [4,5,6] and pharmacology, e.g., for drug delivery. However, it has attracted relatively less support in precision agriculture [7]. We have seen significant progress in fabrication of NPs with unique functions and dimension-based physicochemical characteristics [8]. Owing to their astonishing properties, NPs with sizes 20–100 nm have received greater attention [9]. The sector of nano-enabled products tries, at their scale, to re-evaluate the biomass assets of previously reported anti-pathogenic substances to alter or inhibit the deadly effects of plant pathogens. Appropriate stimulants seem to be NPs with polymers, metals, or porcelain that can show different biological use depending on their intensification.

NPs play a pivotal role in crop development and improving the nutritive benefits in the agricultural field. The NPs can be used as components of novel fertilizers in agriculture [10], substances for crop protection [11], herbicides [12], and preparations for prolonging cut flower durability [13]. NPs and nanomaterials have recently been proposed as potential biostimulators that could help plants propagate and grow faster [14,15] and develop plant resistance against oxidative stress [16]. The use of NPs could provide several advantages to agriculture and horticulture, but it may pose significant hazards due to their unrecognized environmental consequences [17]. However, detection of optimized concentrations of AgNPs for plant disease management without losing the efficiency and while keeping the non-toxicity of AgNPs to other non-target organisms can be achieved. This can be done through rigorous research on AgNPs selection for particular plant or pathogen species and the local conditions. The development and continuous improvement of AgNPs to regulate plant growth, soil quality, diseases, agrochemical usage, and environmental degradation have substantially strengthened soil quality, crop yield, and ecosystem security, which contribute greatly to sustainable farming and agro-ecosystems [18]. The utilization of agricultural inputs to speed up the site-specific monitored supply of nutrients enhances the crop productivity while assuring the minimization of the use of agri-input. This can be done with the help of engineered AgNPs; that is, with or without other agents that have their physicochemical properties. Along with the help for plant security solutions they offer, agro-nanotechnology has been actively improved, and this may ensure increased plant growth. Indeed, in the current and future crop promotion programs, nanotechnology has incredible potential to control pests and diseases.

Different kinds of metal-based NPs have flourished, including copper (Cu), zinc (Zn), titanium (Ti), magnesium (Mg), gold (Au), and silver (Ag). Variously structured AgNPs have been widely utilized for antimicrobial applications and disease prevention for hundreds of years. The physicochemical and structural heterogeneity of AgNPs is generally achieved by manipulating three important parameters: concentrations of reactants, temperature, and pH [19]. In the majority of studies reporting biological synthesis of AgNPs, silver nitrate (AgNO_3_) has been used as the source of silver ions, at 1 mM concentration [20,21]. However, some studies have reported the production of smaller-sized AgNPs when moderate [22] to lower [23] concentrations of AgNO_3_ were used. The temperature is yet another decisive factor that has a significant influence on the time consumed, rate of synthesis, and the stability of the size and properties of AgNPs [24,25]. The pH also control the size and shape of AgNPs, where acidic pH of the reaction medium yields larger-sized AgNPs (Binupriya et al., 2010) than the alkaline pH, as reported for *Epicoccum nigrum* cell filtrate-mediated synthesis of AgNPs [26]. Another example of pH-induced size variability of AgNPs is *Penicillium oxalicum*-mediated fabrication of AgNPs over a pH range of 5–13, where alkaline pH (12) produced lower size (4 nm) of AgNPs, consuming less time for synthesis [27]. This suggests higher and longer-term stability in AgNPs, which could be due to the electrostatic repulsion of ions [27]. Similarly, reaction of AgNO_3_ (1 mM) with *Capsicum annum* extract at pH 9 showed the maximum yield of biologically capped AgNPs [28].

The antimicrobial actions of AgNPs to plant pathogens can be defined as their properties to hinder or eliminate the growth of plant pathogens without damaging neighboring plant tissues [29]. AgNPs appear to be the most intriguing of metal NPs that exhibit adequate biological activity [30]. They have a wide range of beneficial effects on plants at certain concentrations [31] and arrest the growth of associated pathogenic fungi [32]. AgNPs have been shown to boost germination [33], growth exultation [34], vegetative growth [14], shoot induction, proliferation [14], and pigmentation [35]. Generally, three processes including physical, chemical, and biological processes are employed to prepare AgNPs. Physical and chemical approaches are typically found to be costly and environmentally unfriendly. However, biologically produced AgNPs exhibit great yield, good solubility, and excellent stability for prolonged periods. Out of these three, the biological approach has remained as the simplest, environmentally far less toxic, commercially viable and mostly a single step approach that does not require thermal gradients, pressures, forces, or synthetic chemicals [36]. Biosynthesis of AgNPs through plant parts (i.e., leaf or bark or root or stem, or flower extracts) and microorganisms (i.e., from extra or intracellular molecules of fungi, algae, bacteria) is thus a great tool for simplifying their use in agriculture. This review summarizes the recent progress made in the biosynthesis of AgNPs and discusses the potential of AgNPs in various antibacterial, antifungal, antinematic, and antiviral applications from a plant disease perspective.

## 2. Biosynthesis of AgNPs

**Figure 1:**
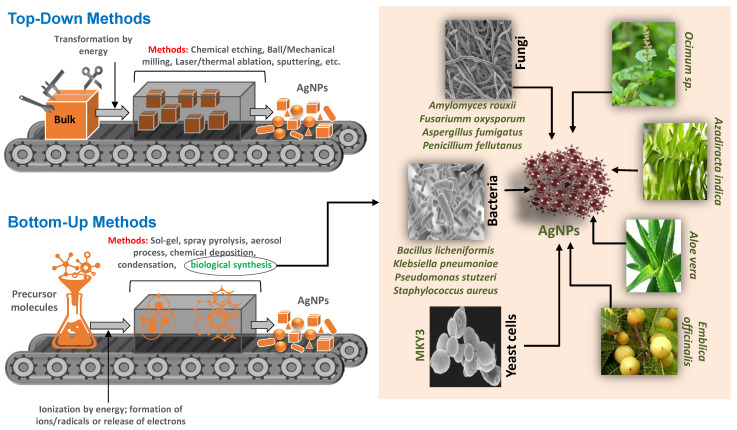
Illustration of two approaches of synthesis of AgNPs in left panel: (i) top-down approaches such as mechanical, chemical, and electro-explosion, etc. In bottom-up approaches, inorganic or organic substances are used as a reducing or capping agents. In bottom-up approaches, various techniques, i.e., superficial fluid synthesis, aerosol process, green synthesis (using bacteria, algae, fungi and plant or their biological products) etc. are used. Right panel shows a few proven examples of microbes and plants for AgNPs synthesis.

There are two primary approaches for the synthesis of NPs, (i) the top-down approach and (ii) the bottom-up approach, which are graphically presented in Figure 1. In the top-down approach, techniques like mechanical milling, etching, electro explosion, sputtering, and laser ablation are used. The bottom-up approach involves the use of superficial fluid synthesis, plasma spraying synthesis, aerosol process, laser pyrolysis, and green synthesis. In green biological methods, plants and microbial constituents are used as essential bio-reducing agents, which also provide versatility in AgNPs synthesis. Biological methods are considered more effective, inexpensive, sustainable, and environmentally non-toxic compared to chemical and physical methods since the biological materials are easily available and require no or less prior processing [37]. Generally, plant and microbially biosynthesized AgNPs are used as a source that can either be processed extracellularly or intracellularly [38]. Moreover, algae, fungi, and beneficial bacteria that can tolerate toxic materials, e.g., heavy metals and organic chemicals, while retaining the capacity of producing organic and inorganic reducing agents may be the biological solution for AgNPs synthesis.

Based on the available literature, a general mechanism of AgNPs formation and growth can be suggested. Functional groups of biomolecules, such as those from polyphenols, release electrons in the reaction medium that interact with the molar concentrations of metal salt (generally AgNO_3_), and reduction of silver ions (Ag^+^) occur. As a result of reduction, particle formation begins and surface plasmon resonance (SPR) increases with a gradual to sharp intensification of dark color of the medium. At some point, the increase in SPR does not increase further and further reduction of Ag+ ions does not occur, which could be due to two major reasons (i) reduction of all available Ag+ ions by reducing agents and/or (ii) lack of adequate electrons in the medium, i.e., low concentration of biomolecules. The reduced atoms of silver (Ag^0^) then start to form aggregates that could be limited to a certain size if the capping agent is present in the reaction medium; otherwise, smaller aggregates further grow and form larger aggregates. The capping of Ag^0^ generally involves Van der Waals forces, electrostatic attraction, or covalent bond formation between capping agents and Ag^0^ atoms, and the AgNPs particle or crystal attains its characteristic size and shape.

### 2.1. Biological Synthesis of AgNPs by Plant Parts

**Figure 2:**
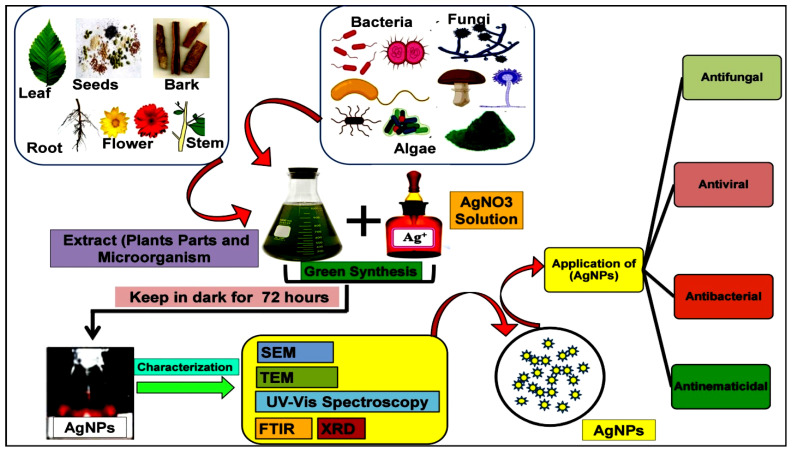
Illustration of green synthesis of AgNPs from various extracts such as plant parts (i.e., stem, leaf, flower, barks, seeds, and root) and microorganisms. Prepared extracts of known dilutions are mixed with predefined molarity of silver nitrate (AgNO_3_) solution and stirred/kept under dark conditions for a fixed time, e.g., 72 h. Following harvest, the AgNPs are physicochemically characterized for size, shape, and surface area, charged by techniques such as transmission electron microscopy (TEM), scanning electron microscopy (SEM), UV-Vis spectroscopy, FTIR, XRD, DLS, and zeta-potential, etc., and applied for several purposes such as removal of bacteria, fungi, viruses, or nematodes.

For the synthesis of AgNPs, plant parts such as leaves, fruits, stems, roots, seeds, and flowers are used due to their abundant quantity and availability, and their biochemical composition is also generally considered non-toxic to humans or other non-target organisms since they are known for producing beneficial phytochemicals. Several plants such as *Acacia nilotica*, *Ocimum sanctum*, *Coleus aromaticus*, *Lantana camara*, and *Moringa oleifera* have been used for AgNPs biosynthesis. Plant-derived AgNPs are characterized by various techniques [28], and based on their properties and need, are implemented in agricultural applications (Table 1 and Figure 2). Plants are widely available as the source of many rich metabolites that serve as bio-agents to reduce the Ag^+^ ions [39]. Biomolecules from several plant species may also be used for the coating of AgNPs in order to reduce the need of chemical reagents and hazardous substances or other approaches for purification [40]. *Eucalyptus globulus* [19], *Azadirachta indica* [41], *Cissus quadrangularis* [42], *Lantana camara* flower [43], *Rubus illecebrosus* leaf [44], *Nephelium lappaceum* peel [45], *Ricinus communis* [46], *Calotropis gigantea* are yet other species used in the green synthesis of AgNPs [47]. Reducing and stabilizing agents in plants help shape biocompatible AgNPs. Available secondary compounds including terpenoids, flavonoids, phenols, alkaloids, proteins, and carbohydrates in the extract function as reduction agents [48,49,50,51].

### 2.2. Biological Synthesis of AgNPs by Bacteria

Biological preparation of AgNPs through microbes has been recognized as a rapidly growing field of research in green nanotechnology. Different microbial entities have constantly been engaged in the synthesis of AgNPs, which are seen as alternatives to the traditional chemical and physical strategies. Many bacterial species such as *Serratia nematodiphila*, *Bacillus stearothermophilus*, *Escherichia coli*, *Morganella morganii* RP42, *Pseudomonas proteolytica*, *P. meridiana*, *B. brevis*, etc. were used in AgNPs biosynthesis (Table 2 and Figure 2). Moreover, several other microorganisms, including *B. methylotrophicus*, *Brevibacterium frigoritolerans*, and *Novosphingobium* sp., have recently been found effective for the production of AgNPs. Mechanistically, bacteria are responsible for secreting different enzymes in broth culture that include several reductases that perform AgNPs biosynthesis [59]. In the process, bacterial cells act as reducing entities because they extracellularly induce the nucleation and capping of NPs by reducing metal ions (Ag^+^) to metal NPs (AgNPs) [60]. Bacteria can offer comprehensive opportunities for the synthesis of AgNPs because of their reasonably easy cultivation process and quick replication efficiency. *Pseudomonas stutzeri* (AG259) was the first bacteria to be used for the synthesis of AgNPs [59]. The AgNPs were also synthesized using *Bacillus* species like *B. megaterium* (NCIM 2326), *B. cereus*, *B. licheniformis*, *B. amyloliquefaciens*, *B. marisflavi*, *B. flexus*, and *B. subtilis* [61].

### 2.3. Biological Synthesis of AgNPs by Algae and Fungi

Among other organisms, algae, a group of marine or freshwater microorganisms, are widely considered as bio-nano factories as both live and dead dried biomasses have been used to synthesize NPs [68]. In addition to algae, fungi also play a significant role in the green synthesis of AgNPs. Algae and fungi serve as reducing agents and function in an environmentally friendly way for sustainable and non-toxic fabrication of AgNPs. The majority of the algae used for this purpose are from the species of *Sargassum plagiophyllum*, *Chlorococcum humicola*, *Amphora*-46, *Caulerpa racemose*, *Padina pavonica*, and *Chaetomorpha linum* etc., and mycelium of fungi, i.e., *Aspergillus fumigates*, *Penicillium fellutanum*, *A. flavus*, *Fusarium semitectum*, and *Alternaria alternata* (Table 3 and Figure 2). Fungal molecules based AgNPs are unique over bacteria in terms of providing higher rates of growth of organism, easy cultivation methods and high levels of extracellular enzyme secretions, increased NPs tolerance (protein coating), and excellent stability with high concentrations of NPs [69]. Massive fungal flora have recently had major research involvement in synthesizing suitable biocompatible AgNPs including *Trichoderma resei*, *A. niger*, *F. oxysporum*, and *Phytophthora infesatans* [70]. For instance, owing to its biologically active substances that have antimicrobial antioxidant, immunomodulating, and anti-cancer properties, *Penicillium oxalicum* have certain benefits in biogenic synthesis [70]. Other fungi like *F. oxysporum*, *F. semitectum*, *F. acuminatum*, *F. solani*, *Cladosporium cladosporioides*, and *T. asperellum* can also synthesize AgNPs with a broad range of applications [71].

## 3. Determining the Size, Shape, and Yield of Biological AgNPs

Determining the shape and size of biologically synthesized AgNPs is the most relevant aspect of AgNPs synthesis since it also determines the interfacial reactions with organisms and intracellular penetration of AgNPs. This can be fine-tuned by optimizing the hydrogen ion concentration of the medium. For example, *Aloe vera* extract mixed at variable pH with solvent resulted in variously sized and shaped Au–Ag core NPs [83]. In most studies, AgNPs have been successfully synthesized at room temperature [84,85], while a few studies also report the control of their shapes by temperature [86]. Regarding shapes, low reaction temperature yielded the formation of nano-triangle AgNPs while high temperature produced spherical AgNPs using *Cymbopogon flexuosus* extract as reducing and capping agent [87]. When plant biomass is used, some physical and chemical parameters control the crystal growth and structure of NPs [88], for instance, the amount/percentage of the metal salt solution. In fact, for near-perfect control over the size and shape of AgNPs, reaction parameters such as concentrations of metal ions and extracts, duration of the reaction, temperature, and pH are adjusted in a series of different reactions [89,90]. Moreover, the biological production of AgNPs is also governed by enzymatic reaction. For example, in a study, Korbekandi et al. showed pre-incubation of *Fusarium oxysporum* with 0.1 mM AgNO_3_ for the activation of reducing enzymes. Additionally, glucose added as electron donor helped in the reduction of AgNPs. The optimized values for four parameters were: 0.1 mM AgNO_3_ as inducer of enzymes, 5 mM AgNO_3_ as substrate, amount of biomass (4.96 g dry weight/L), and 56 mM glucose as electron donor. The use of microbes, algae, and plants for biological synthesis of AgNPs is widely known and has been optimized with several species [91]. However, the green synthesis method is faster and more feasible compared to algae mediated or microbial synthesis. This is possibly due to the easy availability of plant materials that do not require maintenance as in the case of microbes. Plant materials are available in plenty, and since plants have a bigger genome size and more complex physiology, the biomolecular composition of plants is far better in terms of their variety and quicker production of AgNPs. AgNPs produced through green routes are generally more biocompatible and stable [92,93].

## 4. Silver Nanoparticles in Plant Disease Management

### 4.1. AgNPs in Bacterial Disease Management

The antimicrobial properties of AgNPs are significant for a wide range of microorganisms including bacteria, fungi, and viruses [94]. AgNPs measuring 10–100 nm in size have a strong antibacterial effect against several Gram-positive and Gram-negative bacteria [95]. AgNPs are well-known to be effective antimicrobials against multidrug-resistant (MDR) bacteria, such as methicillin-resistant *Staphylococcus aureus*, erythromycin-resistant *Streptococcus pyogens*, ampicillin-resistant *E. coli*, and vancomycin-resistant *S. aureus* [96]. In a study, AgNPs prepared using *Azadirachta indica* extract inhibited the growth of phytopathogens *Penicillium* sp., *Aspergillus* sp., *Fusarium* sp., and *Ralstonia solanacearum* [32]. Silver ions prevent bacterial DNA replication by linking and causing denaturation of the DNA, which involves silver ion interaction with thiol proteins accompanied by DNA condensation, which leads to apoptosis [97]. The antibacterial activity of AgNPs is largely due to the release of silver cations, which can bind bacterial proteins specifically to thiol (-SH) groups and interrupt their physiological functions [98], leading to necrobiosis. AgNPs carry out their antibacterial activities via a Trojan horse mechanism: their preliminary attachment to the surface of the cell leads to modification and degradation of cell membrane, and release of intracellular metal silver ions exerts toxicity [99]. AgNPs showed pronounced inhibition of growth of *E. coli*, *P. aeruginosa*, and *S. aureus* [100]. The antibacterial effects of AgNPs are mostly due to their broad surface layers, which allow for greater contact between the NPs and microbial cells, suppressing cell development at very low concentrations present in surroundings [101]. The weaker susceptibility to NPs of Gram-negative bacteria (i.e., *E. coli*) can even be clarified due to the presence of an external lipopolysaccharide (LPS) coating on their surface that restricts the penetration of compounds. However, elevated concentrations of antibacterial agents are therefore required to achieve the same effect on Gram-positive bacteria [102]. AgNPs rely mostly on size, shape, surface functionalization, and ability to display certain antimicrobial properties [103]. The bactericidal impact is also thought to result from the AgNP-mediated generation of reactive oxygen species (ROS) [104]. Two potential AgNP toxicity mechanisms have been explored [105]. One is that AgNPs have a large bacterial surface contact that can enable the cellular membranes to bind to the particles and penetrate effectively into the bacteria. Another mechanism is the interference of AgNPs or dissolved ions with the respiratory chain of cell membrane. Much evidence demonstrates that the antibacterial activities of AgNPs include silver ions [106,107].

To date, some processes have been demonstrated, including protein disruption [108], ROS production and antioxidant degradation, membrane disability work (e.g., membrane disruption, lack of membrane permeability), mutagenicity, and transport protein gene expression downregulation [109]. Membrane fluidity and metabolic activities are disrupted as a result of the morphological changes of cellular membranes impacted by AgNPs and membrane depolarization, which eventually leads to cell component damage and even death. The leakage of molecules inside the cell, such as enzymes, proteins, metabolites, and DNA, is caused by the breakdown of cellular structures. Furthermore, AgNP can form unusual holes in microbial cell walls, allowing NPs to penetrate extracellular and intracellular areas [110]. TEM may be used to study the behavior of AgNP-induced membrane damage and perforation of cell membranes [98]. In recent studies, TEM shows extensive cell surface disintegration, nuclear and cytoplasmic material leaking, and enlargement of cells [111,112]. Moreover, the antibacterial mechanism of AgNPs can follow a dual mode of action of particles and silver ions [113,114]. Some recent examples of inhibition of growth of bacterial pathogens are listed in Table 4.

### 4.2. AgNPs in Viral Disease Management

In agriculture, AgNPs can be used as antivirals. Viruses are assumed to be involved in almost 50% of all evolving plant diseases, which seem to be difficult to manage; there is no cure in some situations. Viral infections necessitate appropriate and effective management. The viral multiplication cycle includes viral replication into cells, genomic and viral biosynthesis, viral component assembly, and emission of virus particles from the host cell [126]. AgNP’s potential antiviral mechanisms include interactions with gp120, antagonistic impact on virus attachment to the cell, destruction of virus particles before entry, and interaction of AgNPs with double-stranded DNA [127]. AgNPs can also interact with the polypeptide coats of tomato mosaic virus (ToMV) and potato virus Y (PVY) [128]. Resulting from interactions with envelope glycoproteins, AgNPs directly inactivate TMV, causing immediate damage to TMV shell proteins, agglomeration, and even disintegration. Elbeshehy et al. [129], studied AgNPs fabricated from *Bacillus persicus*, *B. pumilus,* and *B. licheniformis*; the AgNPs had an negative influence on the yellow bean mosaic virus. A post-infection treatment given 24 h after virus exposure inhibited the virus from causing any detrimental manifestations. Plants treated with AgNPs and inoculated simultaneously showed only mild BYMV symptoms, and a 72-h pre-infection treatment had almost no influence on viral concentration or disease severity. When AgNPs were applied on *Chenopodium amaranticolor* crops 24 h after treatment, they displayed the most substantial antiviral impact against tomato spot wilt virus (TSWV). Plants sprayed after inoculation had a poor infection, but plants treated before inoculation had a weak inhibition [130]. El-shazly et al. [131] reported that potato plants sprayed with AgNPs 24 h after being inoculated with tomato bushy stunt virus (TBSV) showed a decrease in virus concentration and disease percentage [131]. *Cymopsis tetragonaloba* leaves sprayed with AgNPs and sun hemp rosette virus (SHRV) showed full disease reduction by suppressing virus replication [132]. When compared to individual AgNPs and salicylic acid (SA) treatments, a combination of AgNPs and SA administered before 3 and 7 days of virus infection had a synergistically antiviral impact against TBSV [131]. Because the NPs attached to the virus and hindered viral replication, spraying AgNPs on infected tomatoes for 7 days before treatment with ToMV and PVY reduced disease severity and virus concentration [128].

The expression of antioxidant enzymes POD (peroxidase) and polyphenol peroxidase (PPO) in tomato plants treated with AgNPs and infected with ToMV or PVY increased significantly [128]. Mahfouze et al. [133] discovered that banana plants infected with BBTV and treated with 50 ppm AgNPs did not exhibit any outward symptoms, even though the infection rate was 36%. On the other hand, banana plants treated with 50 ppm AgNPs following viral infection showed a considerable improvement in dry weight and leaf area when compared to BBTV-infected banana plants (viral control). When compared to the healthy control, all plants treated with 50 ppm AgNPs after virus inoculation had considerably higher levels of phenol, proline, and oxidative enzymes. Elbeshehy et al. [129] studied the effect of biosynthesized AgNPs on BYMV-infected fava bean leaves and displayed significant symptoms such as yellow mosaic, mottling, crinkling, size reduction, and deformation, which were not present in non-infected leaves. According to Jain and Kothari [132], AgNPs sprayed over the bean leaves showed 100% suppression of the sun-hemp rosette virus. Cai et al. [134] asserted that antiviral activity of NPs in plants is due not only to inhibition of virus replication, but is also responsible for the stimulation of defense mechanisms in plants that contribute to plant immunity and growth response. For example, AgNPs stimulated a phytohormone cytokinin in *Capsicum annuum* L. [135]. Tripathi et al. [136] suggested that AgNPs adsorption in plants might hinder apoplastic transportation of virus particles by obstructing cell connections, the plasmodesmata, and preventing the apoplastic movement of liquid and nutrients. Hassan et al. [137] employed RAPD and direct amplification of minisatellite-region DNA (DAMD) approaches to detect DNA modification in olive plants exposed to varying concentrations of silver or selenium nanoparticles. They observed that 5 mg L^−1^ AgNPs had a positive effect on in vitro performance. However, 10 mg L^−1^ AgNPs and 2.5 and 5 mg L^−1^ SeNPs induced changes on genomic DNA level. In addition, RAPD and DAMD assays can be successfully applied as a sensitive method of detecting DNA stability and genotoxicity in olive plants treated with different concentrations of selenium and silver NPs. Mahfouze et al. [133] demonstrated that after BBTV infection, the banana plants sprayed with different concentrations of AgNPs showed a few changes at the genomic DNA level, whereas both random amplified polymorphic DNA (RAPD) and sequence-related amplified polymorphism (SRAP) markers scored nearly the same polymorphism in the banana plants (Table 5).

### 4.3. AgNPs in Fungal Disease Management

The utilization of AgNPs as an alternative to synthetic fungicides has been a relatively new approach with more efficacy. AgNPs may be used for the control of plant diseases as part of their implementation. A few studies have been performed so far on the implementation of AgNPs against phytopathogenic fungi [140]. The latest anti-fungal agents are often in demand because of the development of resistance in pathogenic fungi [141]. There have been cases of antifungal resistance globally, which favor the emergence of resistant mutants due to frequent applications of agrochemicals that also alters the fungal population [142,143]. AgNPs synthesized using *A. flavus* inhibited the growth of *Trichoderma* spp. [144]. The AgNPs also showed antifungal properties against *Candida albicans* and *C. tropicalis* [145] and *F. oxysporum* [146]. Due to enhanced permeability and retention effects, the AgNPs seem to be very attractive and fascinating for diverse antifungal applications. Recently, more work has been directed towards developing safe management strategies, which placed humans and animals at lower risk and concentrate on resolving synthetic fungicide vulnerabilities. Nanotechnology represents a way of developing new antifungal secondary metabolites to reduce the symptoms caused by emerging resistant fungi [147]. AgNPs have several antifungal action mechanisms, involving linkages to DNA phosphates groups [148] and plasma–membrane interactions, that contribute to proton dispersion and cell mortality, sulfhydryl protein and enzyme groups, degradation of the electron transportation chain, and disturbance of membrane proton motive force and phosphate groups [149].

AgNPs have been shown to have a wide range of antifungal properties, including against aggressive fungal infections [150] such as *C. albicans*, *C. tropicalis*, *C. parapsilosis*, *C. glabrata* [151], *Trichophyton rubrum* [152], *Trichosporona sahii* [153], *A. niger*, *Rhizoctonia solani*, *Curvularia lunata*, *Colletotrichum* sp., and *Fusarium* sp. [154,155]. DNA impairs the fungal culture’s ability to proliferate after it has been treated with silver crystals (AgCs) [156]; this may result in decreased protein expression in the subunit as well as faulty enzymes and cellular proteins required for ATP generation [157]. Several other plant fungi, such as *Alternaria alternate* and *Trichosporona sahii*, have shown similar effects with AgNPs. The production of reactive oxygen species (ROS) can cause NP toxicity [153]. Free radicals can damage cell walls, proteins, lipids, and DNA, among other biological components. Deletions, mutations, single strand rupture, double-strand breakage, and protein crosslinking are all examples of DNA damage [158] (Table 6).

### 4.4. AgNPs in Nematode Disease Management

Various crops are annually damaged by more than 4100 species of plant pathogenic nematodes [171]. Therefore, the development of new nematicides with lessened side effects on non-target organisms and the environment is critical. Recently, evidence has shown that AgNPs have effective nematicidal potential [172,173]. According to different studies, AgNPs can decrease the nematode infestation through the pretreatment of plant seedlings. Researchers also suggest that AgNPs have a direct toxic effect on nematodes and may result in reproduction inhibition [174]. For this to occur, generation of excessive oxidative stress may be an important mechanism in AgNPs toxicity to nematodes [175]. AgNPs exposure in *Caenorhabditis elegans* decreased reproductive potential and increased ROS formation, expression of the phosphomevalonate kinase (PMK-1/p38), MAPK (Mitogen-Activated Protein Kinases), hypoxia-inducible factor (HIF-1), and glutathione S-transferase (GST) enzyme activity [174]. Degradation in the cell wall of Meloidogyne *incognita* second-stage larvae (J2) was also observed due to AgNP toxicity under lab conditions [176].

A study on micro-crystalline cellulose-embedded AgNPs reported that a dose of 20–40 ppm caused deflection during the growth stages and influenced embryogenesis eggs’ hatchability and survival in J2 of *M. incognita* [173]. In another study, AgNPs synthesized from *Artemisia Judaica* extracts caused an increase in juvenile mortality and egg hatch inhibition in comparison to crude extracts [177]. Furthermore, AgNPs showed antinematic properties against *M. graminicola* in rice without adverse effects on seed germination or plant growth [172]. The anti-nematode primary mode of action of produced AgB-NPs inhibits several physiological pathways in nematode cells such as membrane permeability, ATP organization, and reaction to oxidative stress [174,178]. Structural immunization may be significantly affected by different characteristics such as disparities in cell wall composition and the production of phytochemicals and pathogenesis-related proteins [179]. Furthermore, phytoalexin organization is frequently connected to the resulting resistance stages of produced AgB-NPs [179]. As a response to nanoparticle stimuli, some plants produce a wide range of secondary metabolites, including phenol and other phytochemicals. They may be an important part of the plant’s defense mechanism against insects such as root-knot nematodes [180]. Some studies on AgNPs effects on nematodes are summarized in Table 7.

### 4.5. Mechanisms of AgNPs Mediated Inhibition or Killing of Phytopathogens

Despite plenty of reports on pathogen inhibition by AgNPs, the precise mode of antibacterial/antimicrobial action of AgNPs against phytopathogens is not completely understood. Here, we attempted to gather the available information on common modes of antimicrobial action of AgNPs. The AgNPs show their effects mainly by interacting with the cells as (i) particles and/or (ii) Ag+ ions dissolved from applied AgNPs. Some studies have shown that AgNPs produce excessive intracellular ROS, induce an antioxidant response, and interact with essential macromolecules. For instance, Hajipour et al. [108] reported protein disruption by AgNPs, while Lemire et al. [109] and Xia et al. [153] detected ROS induction by AgNPs and its degradation by cellular antioxidant machinery, which followed destability of membrane, genotoxicity, and inhibition of transporter gene expression. Free radicals (a form of ROS and also a part of free radical chain reaction) disrupts chemical bonding and structure of cell walls and biological molecules, including nucleic acids (i.e., DNA), fats, and proteins. Some of the examples of DNA damage are mutations, deletions, and single/double strand breaks [158]. The toxic outcome of AgNP treatment could bea combination of these effects against phytopathogens, since AgNPs may attack multiple cellular targets at once. This has been explained in many other studies [32,192,193,194]. In the process, AgNPs adhere to the cell membranes through electrostatic attraction, affect the morphology of membranes, and cause membrane depolarization, which makes the membrane more permeable. The AgNPs also inhibit cellular respiration and metabolic activity, leading to direct and/or collateral damage to cell components and induction of cell death. In doing so, the AgNPs overwhelm the microbes and the extensive breakdown of cellular components induces the leakage of essential cell fluids or substances (i.e., essential metabolites, proteins, and enzymes) within and/or outside the cell. The creation holes in membranes and walls of microbes by AgNPs facilitate the release of cell content into intracellular spaces and extracellular environment [110]. This kind of hole or perforation in membrane and on cell surface could be observed by transmission electron microscopy (TEM), and the leakage of cytoplasmic material and swelling was also confirmed by TEM. TEM exhibits severe disruption of cell walls, leakage of nuclear and cytoplasmic material, and swelling [111,112]. Similar effects were observed for the AgNP-treated fungi *Trichosporon asahii* and *Alternaria alternata* [153]. In a bacterium *Azotobacter vinelandii*, AgNPs caused cell membrane damage and were present inside the cells as observed by TEM. These AgNPs induced the formation of hydroxyl radicals in *A. vinelandii* cells that were detected by electron spin resonance [195]. Summarily, the sequence of events of AgNP interactions with phytopathogens can be described as (i) adherence of AgNPs to bacterial cell surfaces based on their charge and size of NPs, (ii) entry of AgNPs to cell interior or dissolution of Ag+ ions from AgNPs, (iii) ROS generation by AgNPs in the inner membrane, (iv) excessive ROS causing oxidative burst and activating cellular components that further induce damage to membranes, (v) subsequent leakage of DNA and other cellular materials leading to cell death.

## 5. Biological AgNPs against Plant Pathogens: Possible Implications in Lab to Land Transfer

Several attempts are being made worldwide by nanotechnologists and microbiologist to improve traditional agriculture via the sustainable use of biologically synthesized AgNPs. The control over size, shape, and yield of AgNPs has been made more advanced due to technological innovations in synthesis and characterization processes of AgNPs. The AgNPs may aid the agricultural industry by combating pathogens and avoiding the plant disease incidences in a somewhat regulated and site-specific manner. In general, application of AgNPs to agriculture have a significant potential to improve agricultural productivity [35]. However, the impacts and fate of applied concentrations of AgNPs on agricultural systems as a whole are not fully defined. Therefore, greater public interest in adopting AgNP-based agricultural products for farming and knowledge of their benefits and issues associated with nano-enabled agricultural products will more likely trigger the awareness and acceptability. Since, nanotechnology is gaining impetus at a faster pace, it may become a crucial and most useful component of agriculture industry, the phototoxicity of synthesized AgNPs could be repressed by coating the powdered AgNPs with biocompatible materials such as polyvinyl pyrrole [196]. The toxicity and use cases of Ag-based nanomaterials and AgNPs have been well-established by the Environmental Protection Agency (EPA) [6,197]. In a report, it was claimed that the formulations of >100 pesticides contain AgNPs, which are incorporated as antimicrobial agents. However, there is a lack of information on their trophic transfer and toxicity to humans. Not all types of AgNPs are expected to be toxic. For example, a citrate-coated “colloidal” AgNP showed no photo-, cyto-, or geno-toxicity to human cells, but its “powder” form was toxic [198]. This could be due to the chemical conversion of powder AgNPs silver oxides or release of silver ions. By using biocompatible materials [198], alternatives can be explored and researched to suppress the unwanted toxicity of AgNPs, and this could be accomplished by engineering AgNPs with biocompatible materials during their growth in the media or after the synthesis. Such biocompatible coatings or materials would certainly increase their effects, such as enhanced germination of seeds and plant growth. Sometimes, environmental factors also affect the morphology and physicochemical characteristics of NPs and may show different effects on different plant species in real soil environment or field conditions [199]. Therefore, one type of AgNPs cannot be the solution for all pathogens and safe for all host plant species. Maximum effectiveness from lab setups (controlled hydroponic, pot media, or soil) to land or field environments could only be achieved if one type of AgNP is optimized for use with one plant species, or a group of related species that face a similar pathogen attack and disease symptoms.

## 6. Environmental Relevance and Possible Toxicity of Biological AgNPs

Despite the well-defined anti-phytopathogenic effects of AgNPs, the harmful impacts or toxicity of AgNPs to non-target organisms or beneficial microbes of the eco-system cannot be denied while they are being used to regulate growth of causal organisms of plant diseases [11,98]. The interaction of AgNPs with organisms may vastly vary in each method of application, i.e., either foliar or soil application. Though the amount of AgNPs applied is low, there is the possibility that they may disperse from the agricultural soil to water and air. The dispersion modes can be surface run-off by rain or leaching to ground water [200]. Therefore, in agricultural applications of AgNPs, the prior knowledge of AgNPs toxicity to other organisms, especially the beneficial rhizoplane/rhizosphere and phylloplane microflora, should be obtained. Additionally, AgNPs have shown toxicity to some edible crop plants [199]. Therefore, only concentrations of AgNPs that are biologically specific to certain phytopathogens, while being safe for other organisms (especially the host plant), should be used.

## 7. Conclusions

Several strategies have been adopted over the last decade to improve the biological synthesis of AgNPs. Increased understanding and utilization of biological materials *viz*. plant parts, bacteria, and fungi have resulted in advantages over conventional approaches. The many reasons for this include eco-acceptability, cost-effectiveness, safety, and pharmaceutical compatibility without the involvement of hazardous chemicals/processes, high pressure, or energy. Biologically synthesized AgNPs provide significant nano-enabled features for broad-spectrum agricultural applications, specifically in plant pathogen inhibition, because they exert antibacterial, antifungal, and antinematic potential. The current review also highlights that AgNPs originating from green routes may also accumulate in the ecological niche; however, their expected risk to the microbiome and plants is variable.

Various green methods have been established for the production of anti-plant pathogen AgNPs with high yield, purity, and control over their shapes and sizes. Further research should be directed towards the selection of threshold concentrations of AgNPs that efficiently minimize disease instance or severity in plants without exhibiting detrimental impact on the non-target organisms and soil fertility. The physicochemical nature of produced AgNPs can be specific to one crop species and local environmental conditions.

## Figures and Tables

**Table 1:** Biological synthesis of AgNPs from various plant species.

Plants	Family	Plant Part	Metabolites and Their Structures			Corresponding Particle Size	Refs.
*Acacia nilotica*	Fabaceae	Pod	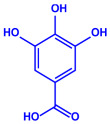		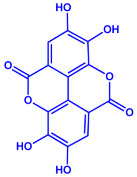	20–30 nm	[52]
			Gallic acid (C_7_H_6_O_5_)		Ellagic acid (C_14_H_6_O_8_)		
			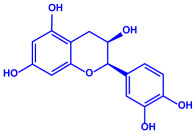		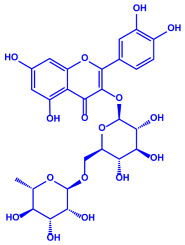		
			Epicatechin (C_15_H_14_O_6_)		Rutin (C_27_H_30_O_16_)		
*Ocimum sanctum*	Lamiaceae	Fresh leaves	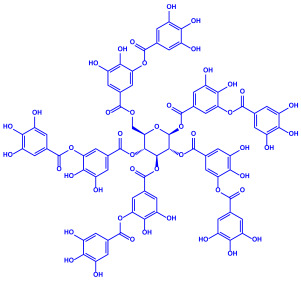			15.72 nm	[53]
			Tannins (tannic acid; C_76_H_52_O_46_)				
			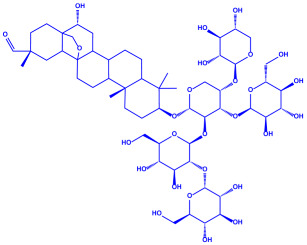				
			Saponin (C_58_H_94_O_27_)				
*Coleus aromaticus*	Lamiaceae	Fresh leaves	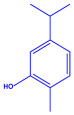	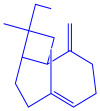	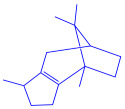	44 nm	[54]
			Carvacrol (C_10_H_14_O)	Caryophyllen (C_15_H_24_)	Patchoulene (C_15_H_24_)		
*Lantana camara*	Verbenaceae	Fresh leaves	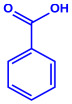	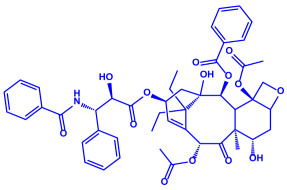		14–27 nm	[55]
			Phenolic acid	Terpenoid			
			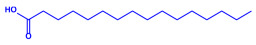				
			Lipid				
			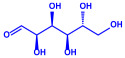				
			Carbohydrate				
*Piper longum*	Piperaceae	Dried fruit	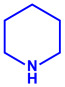	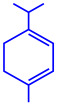	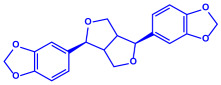	15–200 nm	[56]
			Piperidine (C_5_H_11_N)	Terpinenes (C_10_H_16_)	Sesamin (C_20_H_18_O_6_)		
*Moringa oleifera*	Moringaceae	Fresh stem bark	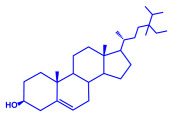		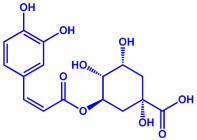	40 nm	[57]
			β-sitosterol (C_29_H_50_O)		Caffeoylquinic acid (C_16_H_18_O_9_)		
*Syzygium cumini*	Myrtaceae	Air dried seeds	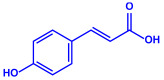		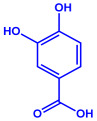	40–100 nm	[58]
			p-coumaric acid (C_9_H_8_O_3_)		3,4-dihyroxybenzoic acid (C_7_H_6_O_4_)		

**Table 2:** Biological synthesis of AgNPs by various bacterial strains.

Bacterial Strains	Metabolites	Structure			Size	References
*Serratia nematodiphila*	(**a**) Prodigiosin (**b**) Sodorifen (**c**) p-Nitrophenol, etc.	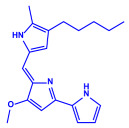	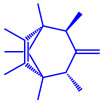	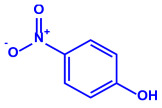	65–70 nm	[61]
		Prodigiosin	Sodorifen	p-Nitrophenol		
*Bacillus stearothermophilus*	(**a**) Macrolactin-A (**b**) Bacillibactin, etc.	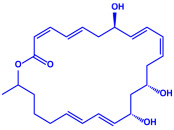			14 nm	[62]
		Macrolactin-A				
		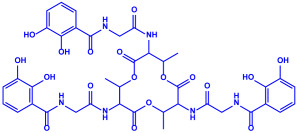				
		Bacillibactin				
*Bacillus strain* CS11	(**a**) Bacteriocin (**b**) Surfactin, etc.	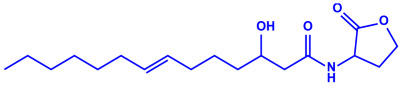			42–92 nm	[63]
		Bacteriocin (small)				
		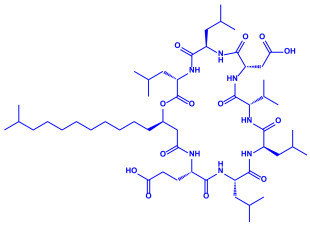				
		Surfactin				
*Escherichia coli*	(**a**) 13-Tetradecynoic acid (**b**) Hexadecanol, etc.	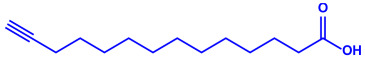			NA	[64]
		13-Tetradecynoic acid				
		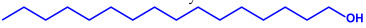				
		Hexadecanol				
*Morganella morganii* RP42	(**a**) Phenyl-β-d-glucoside (**b**) Phenyl ester, etc.	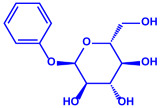		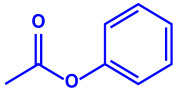	10–50 nm	[65]
		Phenyl-β-D-glucoside		Phenyl ester		
*Pseudomonas proteolytica* and *Pseudomonas meridiana*	(**a**) Phenazines (**b**) Hydrogen cyanide (**c**) Pseudomonine, etc.	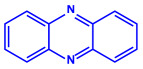		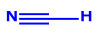	4–13 nm	[66]
		Phenazine		Hydrogen cyanide		
		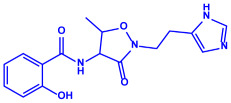				
		Pseudomonine				
*Bacillus brevis* (NCIM 2533)	(**a**) Bacilotetrin (**b**) Hetiamacin, etc.	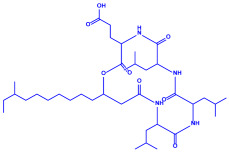		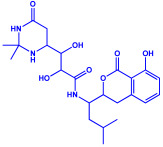	68 nm	[67]
		Bacilotetrin		Hetiamacin		

**Table 3:** Biological synthesis of AgNPs by various strains of algae and fungi.

Algae	Metabolites	Structure		Size	References
*Sargassum plagiophyllum*	(**a**) Saponins (**b**) Anthocyanins (**c**) Triterpenes, etc.	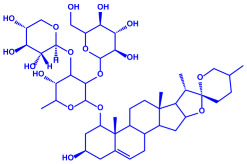	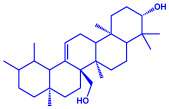	18–42 nm	[64]
		Saponin	Anthocyanin		
		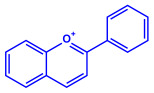			
		Triterpene			
*Chlorococcum humicola*	(**a**) Anthraquinone (**b**) Steroids (**c**) 1-propene, etc.	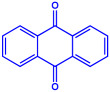	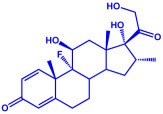	4–6 nm	[72]
		Anthraquinone	1-propene		
		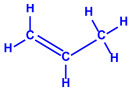			
		Steroid			
*Amphora*-46	(**a**) β-carotene (**b**) Catechin (**c**) p-coumaric acid, etc.	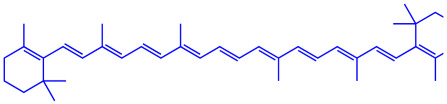		5–70 nm	[73]
		β-carotene			
		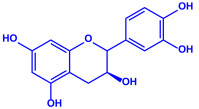			
		Catechin			
		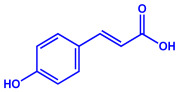			
		p-coumaric acid			
*Caulerpa racemose*	(**a**) α-tocopherol (**b**) β-sitosterol, etc.	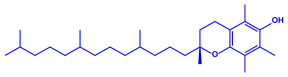		5–25 nm	[74]
		α-tocopherol			
		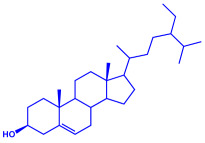			
		β-sitosterol			
*Padina pavonica*	(**a**) Ferulic acid (**b**) Naringenin (**c**) Kaempferol, etc.	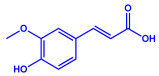	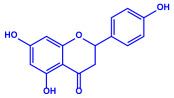	10–72 nm	[75]
		Ferulic acid	Naringenin		
		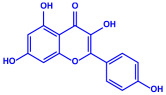			
		Kaempferol			
*Chaetomorpha linum*	(**a**) Linoleic acid (**b**) Arachidonic acid (**c**) Eicosapentaenoic acid, etc.	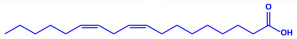		3–44 nm	[76]
		Linoleic acid			
		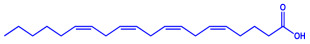			
		Arachidonic acid			
		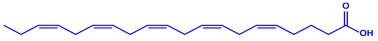			
		Eicosapentaenoic acid			
*Gelidiumamansii*	(**a**) Lactic acid (**b**) Butyric acid, etc.	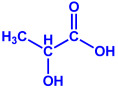	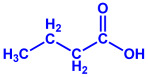	27–54 nm	[77]
		Lactic acid	Butyric acid		
**Fungi**					
*Aspergillus fumigates*	(**a**) Fumigaclavine (**b**) Fumagillins, etc.	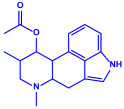		5–25 nm	[78]
		Fumigaclavine			
		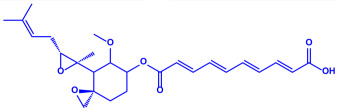			
		Fumagillins			
*Penicillium fellutanum*	(**a**) Fellutamide (**b**) Citrinin, etc	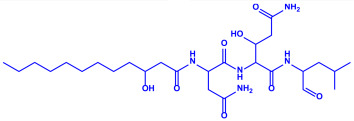		5–25 nm	[79]
		Fellutamide			
		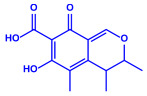			
		Citrinin			
*Aspergillus flavus*	(**a**) Aflavarin (**b**) Cladosporin, etc.	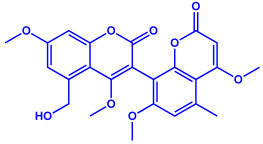		8.92 nm	[80]
		Aflavarin			
		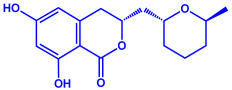			
		Cladosporin			
*Fusarium semitectum*	(**a**) Fusapyrone (**b**) Deoxyfusapyrone, etc.	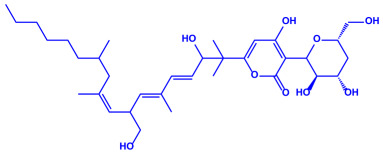		10–60 nm	[81]
		Fusapyrone			
		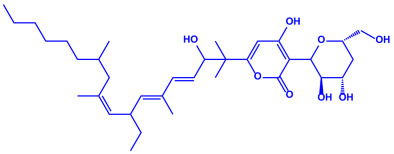			
		Deoxyfusapyrone			
*Alternaria alternata*	(**a**) Tenuazonic acid (**b**) Maculosin, etc.	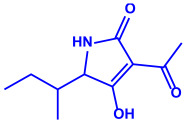	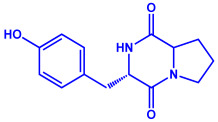	20–60 nm	[82]
		Tenuazonic acid	Maculosin		

**Table 4:** AgNPs in bacterial disease management.

AgNP Types	Size (nm)	Pathogen(s)	Effect(s)	References
AgNPs	25 to 50	*Xanthomonas oryzae* pv. *Oryzae* strain LND0005 and *Acidovorax oryzae* strain RS-1	Inhibited bacterial growth, biofilm formation	[115]
AgNPs	-	*Bacillus subtilis* and *Escherichia coli*	Suppressed the growth of pathogens	[116]
AgNPs	7 and 25	*Edwardsiella tarda* and *E. coli*	Antibacterial activity	[117]
AgNPs	27	*P. aeruginosa* and *S. marcescens.*	Antibacterial activity	[117]
AgNPs	-	*Klebsiella pneumoniae*, *P. aeruginosa*, *S. marcescens*, *Streptococcus pyogenes*, and *Staphylococcus aureus*	In vitro activity against bacterial pathogens	[118]
AgNPs	-	*E. coli*	Suppressed biofilm formation	[119]
AgNPs	12	*Clavibacter michiganensis* subsp. *michiganensis (Cmm)*	Inhibited bacterial canker in tomatoes	[120]
AgCSs	15 to 25	*X. oryzae* pv. *oryzae*	In vitro activity against blight disease of rice	[121]
AgNPs	24.5	*R. solanacearum* strain YY06	Negatively affected bacterial growth, biofilm formation, swimming motility, induced cell membrane damage, and reactive oxygen species (ROS)	[122]
AgNPs	25 to 75	*E. coli* and *S. aureus*	Applied as antibacterial material for fruit and vegetable preservation	[123]
AgNPs	18 to 39	*X. oryzae pv. oryzae*	Increased the plant biomass with a decreased levels of cellular ROS	[124]
AgNPs	470	*X. phaseoli* pv. *phaseoli*	Growth inhibition	[125]

**Table 5:** AgNPs in viral disease management.

AgNP Types	Size (nm)	Plant	Pathogen	Effect(s)	References
AgNPs	10–20	*Cymopsis tetragonaloba*	Sunhemp Rosette Virus (SHRV)	Complete suppression of the disease	[132]
AgNPs	77	*Vicia faba*	Bean Yellow Mosaic Virus (BYMV)	Decrease in virus concentration, percentage of infection and disease severity, reduction in lesions on infected leaves	[129]
AgNPs	12	*Solanum tuberosum*	Potato Virus Y (PVY)	Resistance to virus infection	[131]
Graphene oxide-silver NPs (GO-AgNPs)	30–50	*Lactuca sativa*	Tomato Bushy Stunt Virus (TBSV)	Decrease in virus concentration, infection percentage, and disease severity	[138]
AgNPs	12.6	*Solanum tuberosum* L. *cv. Spunta*	Tomato Spotted Wilt Virus (TSWV)	Decrease in TSWV infectivity and produces an inhibitory effect in local lesions	[130]
AgNPs	-	*Solanum lycopersicum*	Tomato Mosaic Virus (ToMV) Potato Virus Y (PVY)	Reduction in disease severity and virus infection	[128]
AgNPs	-	Autotrophic plants	Banana Bunchy Top Virus (BBTV)	Inhibition of apoplastic trafficking by blocking pores and barriers in the cell wall or plasmodesmata	[136]
AgNPs		*S. tuberosum*	Potato Virus Y (PVY)	Induced resistance to virus	[138]
Schiff base AgNPs		*N. tabacum*	Tobacco Mosaic Virus (TMV)	Induced resistance to virus by promoting plant immunity	[139]

**Table 6:** AgNPs in fungal disease management.

Nanoparticles	Size (nm)	Pathogen	Effect	References
AgNPs	50.6	*Helminthosporium rostratum*, *Fusarium solani*, *F. oxysporum* and *Alternaria alternata*	Effectively mitigated the mycelial growth	[159]
AgNPs	10–12	*F. chlamydosporum* and *Aspergillus flavus*	Suppressed the growth of pathogens	[160]
OT-AgNPs	5–61	*F. oxysporum*, *A. niger*, and *A. flavus*	Antifungal ability	[161]
AgNPs	15	*Candida albicans*	Suppressed the growth of pathogens	[162]
AgNPs	25.6	*A. terreus* Thom	Retardation in fungus growth and biomass	[163]
AA.AgNPs and SD.AgNPs	8–52 and 5–45	*A. niger*, *A. flavus* and *F. oxysporum*	Highly antifungal effect against pathogens	[164]
AgNPs	-	*F. oxysporum*	Antifungal activity	[165]
AgCSs	-	*Aspergillus* sp.	Abnormal spore germination and distorted hyphae	[166]
AgNPs	47	*Colletotrichum gloeosporioides*	Controlled black anther infection during storage of cut orchid flowers	[167]
MC.AgNPs and PG.AgNPs	5–29 and 5–53	*A. niger*, *A. flavus* and *F. oxysporum*	Inhibitory action	[168]
AgNPs		*A. fumigates*, *A. niger*, *A. flavus*, *Trichophyton rubrum*, *C. albicans*, and *Penicillium* sp.	Inhibition of fungal growth and biofilm	[169]
AgNPs	100	*M. phaseolina*, *S. sclerotiorum*, and *D. longicolla*	Inhibited the growth of fungi	[170]

**Table 7:** AgNPs in nematode disease management.

Nanoparticles	Size (nm)	Target Nematode	Test Crop	Effect	References
AgNPs	100	*Heterodera sacchari*	*Oryza sativa*	Decreased nematode population in the root and soil, improved vegetative development of the rice plant	[181]
AgNPs	15	*Meloidogyne incognita*	*Solanum nigrum*	Apart from nematode movement, impacts on production, embryogenesis, hatchability percentage, and larval stages were evident	[173]
Et-AgNPs	20–30	*M. incognita*	*S. lycopersicum*	Inhibition of J2 worms and prevention of egg hatching (in vitro). In vivo infestation of tomato roots was considerably decreased when a root dip therapy with AgNPs was used	[182]
AgNPs	30–100	*M. incognita*	*S. melongena*	Inhibition of eggs and 2nd juvenile (J2) stage of *M. incognita*	[183]
AgNPs	2	*M. incognita*	*Arachis hypogea*	Vegetative growth and fruit weight were increased to varying degrees when the nematode population was diminished	[181]
AgNPs	50–150	*M. incognita*	*S. lycopersicum*	Antagonistic effect on the nematode eggs and larval stages	[177]
AgNPs	5–50	*M. incognita*	*S. lycopersicum*	Highest increase in growth parameters, as well as the minimum galls and egg masses	[184]
AgNPs	20	*M. graminicola*	*O. sativa*	A substantial reduction in the formation of root galls	[172]
AgNPs	16	*M. javanica*	Faba bean	Drastically decreased egg hatching, increased larval mortality, diminished root galling, and J2 population in soils	[185]
Green Silver Nanoparticles (GSN)	8–19	*M. javanica*	*S. melongena*	Reduced second-stage juveniles (J2s), nematode population in soil, and enhanced growth characteristics	[186]
AgNPs	-	*M. incognita*	Bermuda grass	Increased turfgrass productivity in one year and reduced root gall development in two years without phytotoxicity	[187]
AgNPs	5–10	*M. incognita*	*S. lycopersicum*	Significant reduction in the number of galls, egg masses, developmental stage, rate of build up, and nematode population in soil	[176]
AgNPs	13.09 and 10.51	*M. javanica*	*S. lycopersicum*	Increased the plant defense gene’s expression (chitinase gene)	[188]
AgNPs	20	*M. incognita*	*S. lycopersicum*	Second-stage juvenile immobility and mortality	[189]
Ag-BNPs	29.55	*M. incognita*	*S. lycopersicum*	Reduced the level of second-stage juveniles, females, and developmental stages while improving the host plant’s resistance and immunity	[190]
AgNPs	25 to 55	*M. incognita*	*S. lycopersicum*	Galls, egg masses, females per root system/plant, and juvenile mortality were all reduced, and the immune system was induced to resist against nematode infection	[191]

## Data Availability

Not applicable.
